# Iatrogenic Coronary Dissection: From an Unexpected Diagnosis to a Lifesaving Intervention

**DOI:** 10.7759/cureus.95431

**Published:** 2025-10-26

**Authors:** Driss Britel, Khaoula Benslimane, Soufiane Touiti, Meriem El Marzouki, Zouhair Lakhal, Aatif Benyass

**Affiliations:** 1 Cardiac Catheterization Unit, Military Hospital Mohamed V, Rabat, MAR; 2 Cardiac Intensive Care Unit, Military Hospital Mohamed V, Rabat, MAR

**Keywords:** coronary artery angiography, iatrogenic coronary dissection, life-threatening complications, primary angioplasty, st-elevation myocardial infarction (stemi)

## Abstract

Iatrogenic coronary dissection occurs when the wall of a coronary artery is mechanically ruptured during coronary angiography or angioplasty. Although this complication is uncommon, it can be dangerous and may rapidly progress to a life-threatening condition. We describe the successful thrombolytic treatment of a 62-year-old ex-smoker who was admitted with an antero-septo-apical ST-elevation myocardial infarction. During the procedure, an extra backup 3.5 guiding catheter was inadvertently engaged in the ostium of the right coronary artery (RCA), causing an iatrogenic dissection that required immediate stenting, as confirmed by follow-up coronary angiography. This case illustrates the challenges in diagnosing and managing this condition. While angiography remains the primary diagnostic tool for identifying the intimal flap, treatment should be tailored to the patient’s clinical presentation. Management options range from simple observation to stenting or, in severe cases, bypass surgery. The RCA is particularly susceptible to this complication because of its anatomical features and risk factors, such as female sex and deep intubation. This case underscores the importance of meticulous technique during endovascular procedures and maintaining a high level of vigilance for any unusual post-procedural presentation.

## Introduction

Iatrogenic coronary dissection (ICD) is a rare but potentially fatal complication of diagnostic coronary angiography and percutaneous coronary intervention (PCI). Although its incidence is low, it represents a true cardiovascular emergency, as it can abruptly compromise coronary blood flow, leading to acute myocardial ischemia or infarction. Early recognition and prompt management are therefore essential to prevent catastrophic outcomes.

From a pathophysiological standpoint, ICD results from mechanical trauma to the arterial wall caused by endovascular devices such as catheters, guidewires, or balloons. This injury disrupts the intima, allowing blood to enter the subintimal or medial layers and create a false lumen that may compress the true lumen. The dissection can propagate both distally and retrogradely along the vessel wall, potentially extending to the aortic root and resulting in secondary aortic dissection. The hemodynamic impact depends on the degree of luminal compromise, which may range from minimal flow disturbance to complete coronary occlusion with cardiogenic shock. The right coronary artery (RCA) is involved in nearly 85% of cases, likely due to its tortuous course and acute takeoff angle from the aorta [1].

Beyond its procedural context, ICD shares pathophysiological features with spontaneous coronary artery dissection and may occasionally mimic clinical entities such as myocardial infarction with nonobstructive coronary arteries, underscoring the importance of careful angiographic assessment and intravascular imaging [2]. Understanding these mechanisms is crucial for differentiating iatrogenic dissections from other causes of coronary injury and for guiding appropriate therapeutic strategies.

In this report, we present a representative case of ICD occurring during coronary catheterization. Through this case, we aim to highlight predisposing factors, characteristic clinical and angiographic findings, and optimal management strategies, supported by a review of the current literature.

## Case presentation

We present the case of a 62-year-old man, a former smoker who had quit 15 years earlier, his only identifiable modifiable cardiovascular risk factor. The patient had no significant comorbidities and was admitted to the emergency department of Military Hospital Mohamed V with chest pain suggestive of acute coronary syndrome.

**Figure 1:**
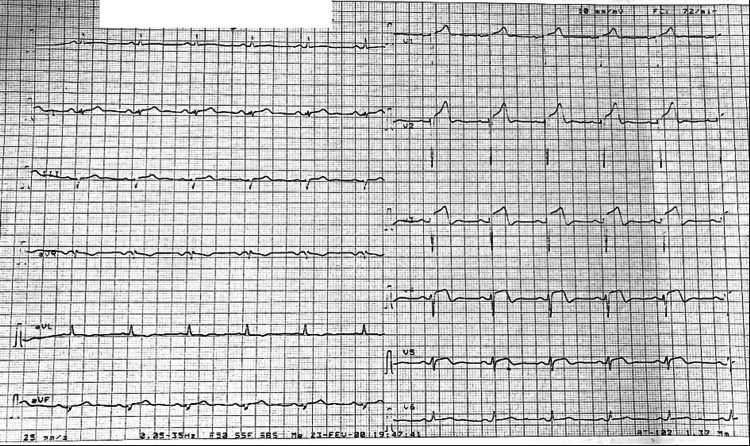
ECG performed at six hours and 30 minutes revealing ST-segment elevation in the antero-septo-apical territory (prior to thrombolysis)

Upon admission, he was hemodynamically stable, with a blood pressure of 165/75 mmHg (equal in both arms), a regular heart rate of 75 bpm, and an oxygen saturation of 97% on room air. Cardiac auscultation revealed no murmurs or additional sounds, while pulmonary examination identified bilateral basal crackles. The ECG showed ST-segment elevation, reaching up to 4 mm, with Q waves indicating necrosis in the antero-septo-apical territory (Figure 1).

**Figure 2:**
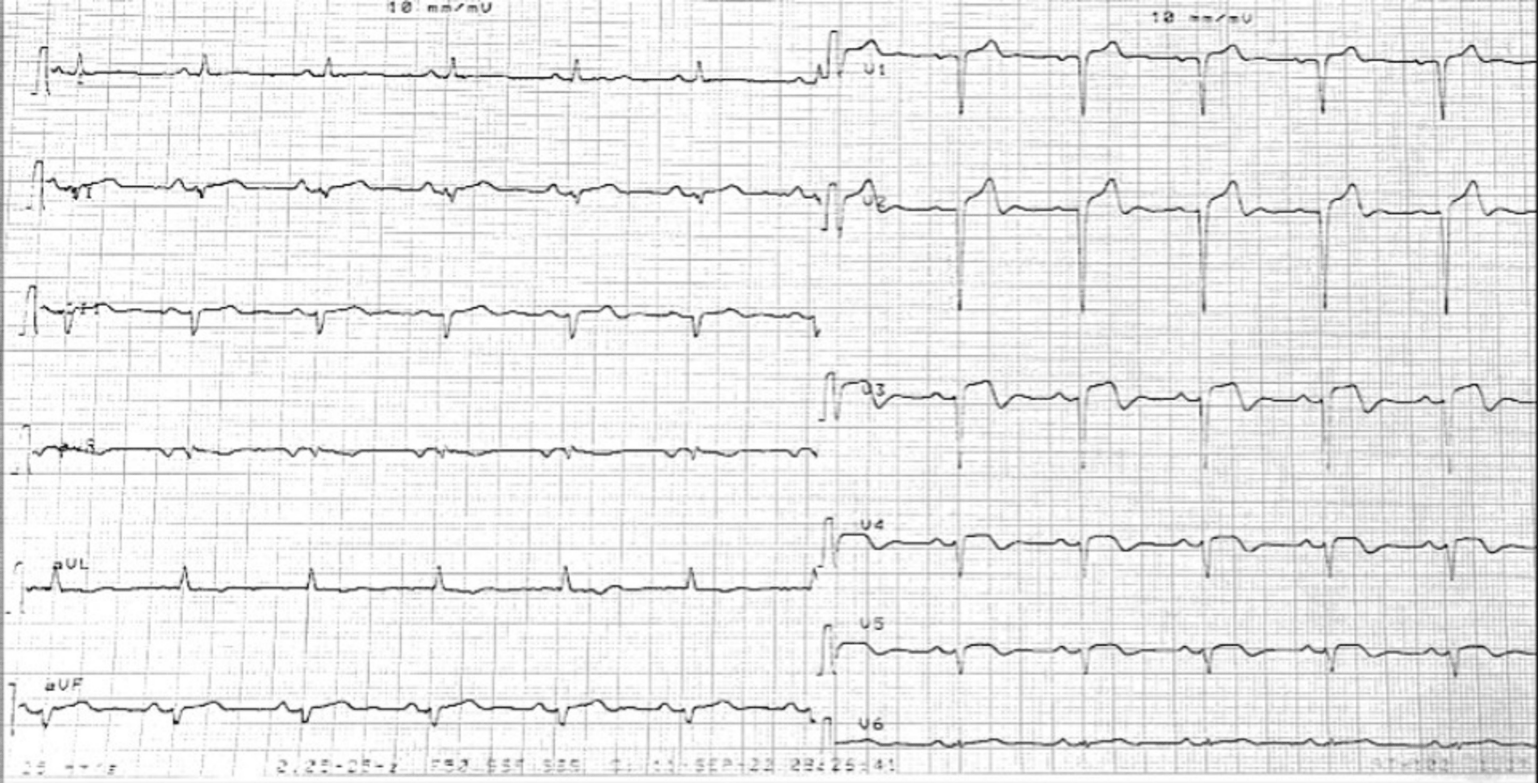
ECG performed two hours after thrombolysis revealing regression of ST-segment elevation

Given persistent pain six and a half hours after symptom onset and the absence of contraindications, thrombolytic therapy was initiated following the administration of aspirin (300 mg) and clopidogrel (600 mg), in anticipation of early pharmaco-invasive angiography. Post-thrombolysis, the patient exhibited clinical improvement, with a visual analog pain score of 3/10, electrocardiographic regression of ST-segment elevation by more than 50% (Figure 2), and evidence of reperfusion, including an accelerated idioventricular rhythm and frequent ventricular extrasystoles.

**Figure 3:**
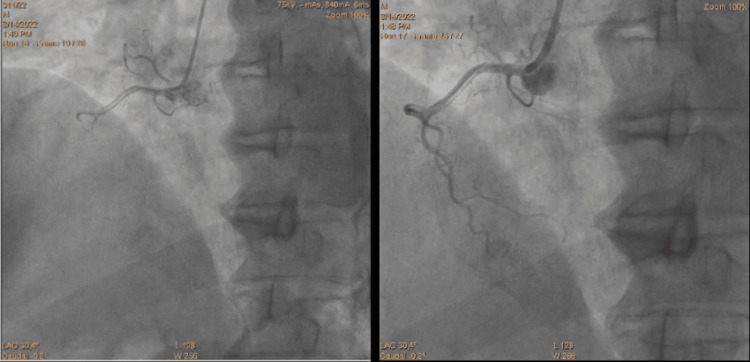
Coronary angiography showing an iatrogenic dissection of the RCA RCA, right coronary artery

**Figure 4:**
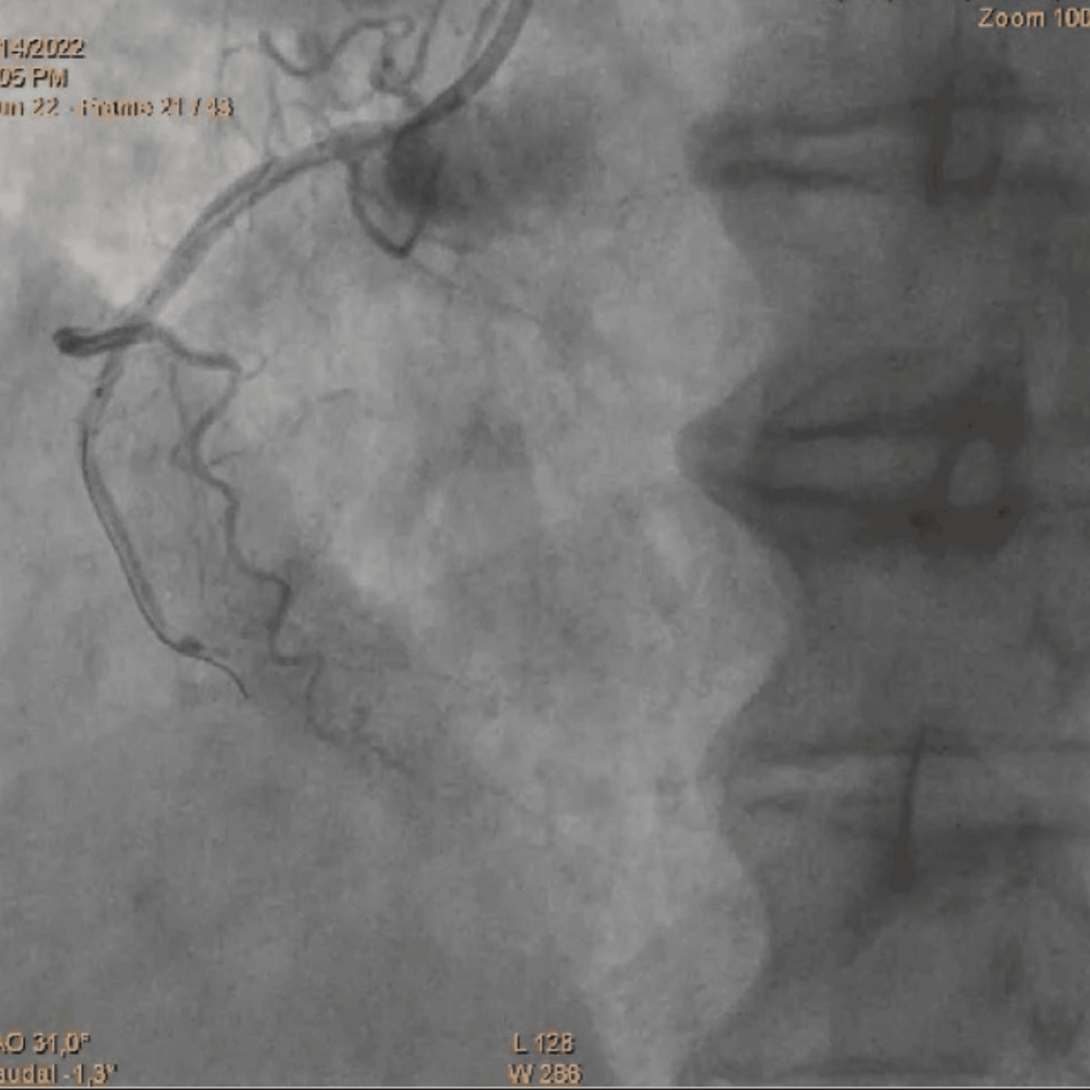
Coronary angiography showing successful passage of the guidewire into the true lumen of the iatrogenic dissection of the RCA RCA, right coronary artery

**Figure 5:**
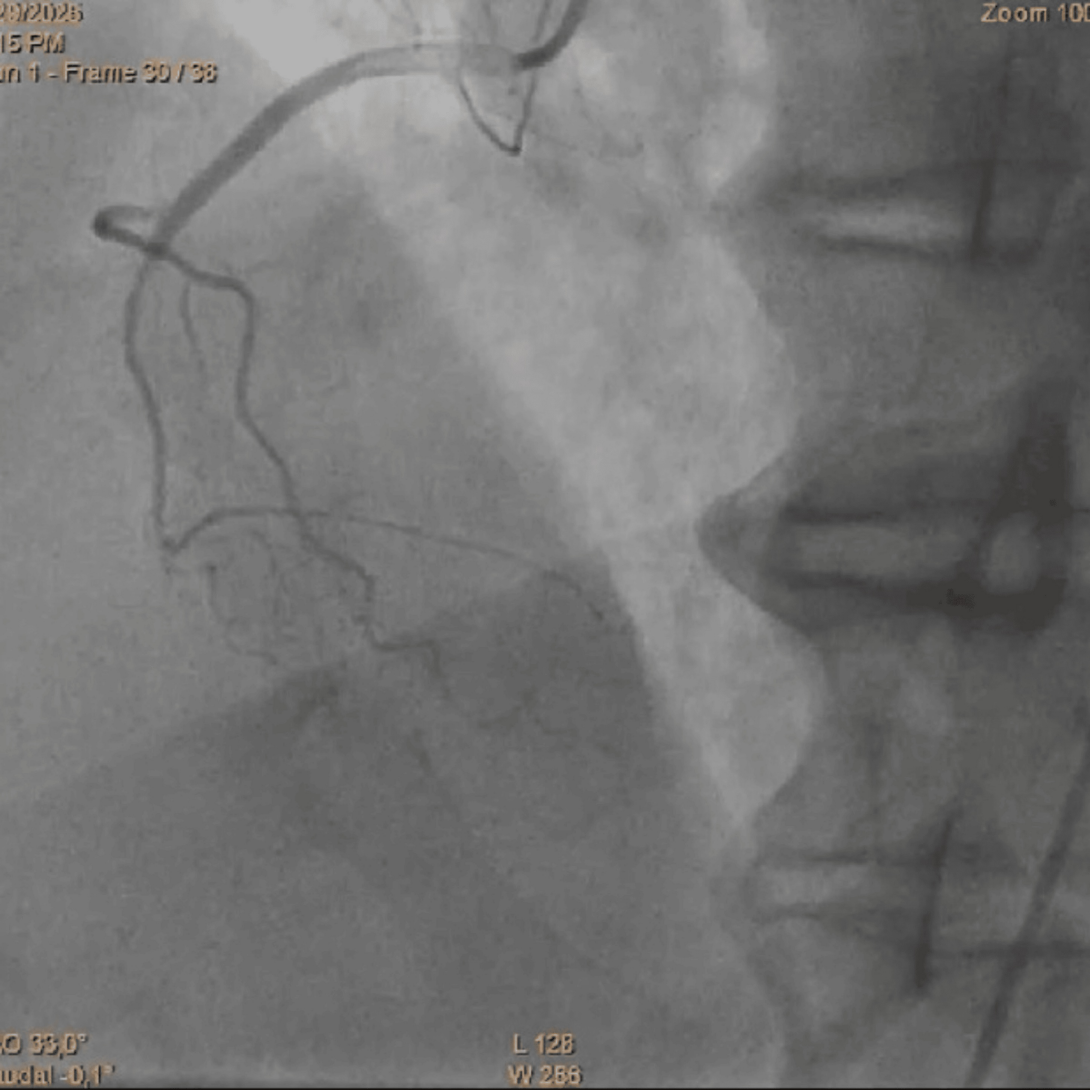
Final result after angioplasty of the RCA RCA, right coronary artery

A control coronary angiogram performed on Day 1 revealed critical stenosis of the mid-left anterior descending (LAD) artery, which was successfully treated with angioplasty and implantation of a drug-eluting stent. The left circumflex and right coronary arteries were initially free of significant disease. Incidentally, a spiral-type iatrogenic dissection of the RCA was identified, secondary to inadvertent insertion of an extra backup (EBU) 3.5 guiding catheter into the RCA. This led to intermittent coronary flow, hemodynamic instability, and a visible false lumen during contrast injection. The dissection was classified as type D according to the National Heart, Lung, and Blood Institute (NHLBI) classification, and urgent stenting of the RCA (2.25 × 35 mm drug-eluting stent) achieved complete exclusion of the false lumen and restoration of TIMI 3 flow (Figure 3, Figure 4, Figure 5).

The patient received a 12-hour tirofiban infusion after PCI due to angiographic evidence of thrombus burden and transient slow flow. The therapy was administered under close clinical and laboratory monitoring and was not associated with any bleeding events. Unfractionated heparin was continued for 24 hours, while dual antiplatelet therapy with aspirin and clopidogrel was maintained thereafter. Laboratory tests revealed moderate renal impairment, pyuria with neutrophilic predominance, elevated C-reactive protein (4.8 mg/L), markedly increased troponin levels (323,000 ng/mL), and an NT-proBNP level of 5,800 pg/mL. No previously undiagnosed diabetes or dyslipidemia was detected. Echocardiography demonstrated elevated filling pressures, a left ventricular ejection fraction of 40%, and regional wall motion abnormalities in the LAD territory.

After several days of observation and clinical improvement under appropriate diuretic therapy, the patient was discharged from the intensive care unit and subsequently from the hospital ward in stable condition.

## Discussion

ICD is a rare complication, occurring in less than 0.1% of combined diagnostic coronary angiography and angioplasty procedures [2]. However, most documented cases are associated with angioplasty, particularly in the context of chronic total coronary occlusion [3]. The increased risk is attributed to the use of large-caliber guidewires, catheters, and other intracoronary devices [4]. Additional reported risk factors include female sex [5], complex lesions, deep intubation, use of Amplatz-type catheters, infiltration of the left main trunk, and contrast injection during deep expiration [6].

These factors did not apply to our patient, a man who underwent a straightforward diagnostic coronary angiography using a JR 4.0 5F catheter, with simple and coaxial RCA intubation.

According to studies by Yip et al. [1] and Ramasamy et al. [7] (a 10-year cohort of 55,968 patients), the RCA is most commonly affected by ICD, compared with the left main trunk (85% vs. <15% and 50% vs. 45%, respectively). Moreover, the ICD of the RCA is more likely to extend retrogradely than dissections involving the left main trunk. This discrepancy may be explained by the denser matrix of type I collagen fibers and higher concentration of smooth muscle cells in the periosteal wall and sinotubular junction of the left main trunk [8]. The risk of ICD is also significantly increased when the RCA is catheterized using EBU or Amplatz left catheters, which were originally designed for the left coronary system [9].

Dissection typically originates at the site of an atherosclerotic plaque, and catheter-induced plaque rupture can propagate due to pulsatile blood flow [10]. Several classification systems have been proposed: the NHLBI classification for coronary dissections [10], the Dunning classification for retrograde extensions [11], and Eshtehardi’s classification for left main trunk dissections [12].

The diagnosis of ICD should be suspected in any patient who develops clinical deterioration in the catheterization laboratory, particularly new-onset chest pain or cardiogenic shock. Some patients, however, may remain completely asymptomatic [8]. ST-segment elevation on the monitor indicates coronary occlusion [8], while arrhythmias or conduction disturbances may also occur, as seen in our patient. Coronary angiography provides a definitive diagnosis, revealing a dissection flap with a contrast defect or stagnation, sometimes accompanied by interruption of coronary flow [3]. Intravascular ultrasound can assist in evaluating the retrograde extension of ICD and in guiding stent placement [13,14]. In contrast, optical coherence tomography should be avoided because the contrast injection may exacerbate the dissection [3]. Thoracic computed tomography remains the gold standard for identifying associated aortic dissection [3].

Effective management requires both technical expertise and composure. Most patients can be successfully treated if the operator remains calm, stabilizes the catheter, and acts promptly [15]. The therapeutic approach depends on the extent of the dissection and the patient’s hemodynamic condition. Conservative management may be appropriate for benign dissections, while surgery or stenting is required in more severe cases. Conservative treatment is generally recommended for mild dissections (Types A and B according to the NHLBI classification) if the angiographic appearance remains stable after ten minutes [16]. Invasive management is indicated for Types C to F dissections because of their poor prognosis [16] and frequent disruption of coronary flow. After advancing a soft guidewire into the true lumen, stenting should be performed to seal the entry point, with careful monitoring of contrast injection, especially in hemodynamically unstable patients [17]. However, the risk of in-stent restenosis is considerably higher when stenting is employed [18]. Coronary artery bypass grafting is reserved for stable patients with aortic root extension or when guidewire passage into the true lumen is unsuccessful [17].

Prevention relies on meticulous catheterization technique, appropriate selection of guidewires and catheters, avoidance of deep intubation, and refraining from injections when the pressure waveform is dampened [4].

## Conclusions

ICD is a rare but potentially fatal complication of coronary catheterization that requires prompt recognition and immediate management to prevent irreversible myocardial damage or aortic extension. This case highlights the critical importance of maintaining vigilance during catheter manipulation, particularly in the RCA, and recognizing angiographic signs of dissection at an early stage. Optimal outcomes depend on an individualized therapeutic approach based on the type of dissection and the patient’s hemodynamic status, ranging from conservative management to urgent stenting or surgery. Prevention remains paramount and involves careful catheter selection, avoidance of deep intubation, and meticulous contrast injection technique. Continuous operator training, familiarity with anatomical variations, and adherence to best practices are essential to minimizing the risk of this serious iatrogenic complication and enhancing procedural safety in interventional cardiology.
